# The *FAD2* Gene in Plants: Occurrence, Regulation, and Role

**DOI:** 10.3389/fpls.2017.01789

**Published:** 2017-10-18

**Authors:** Aejaz A. Dar, Abhikshit R. Choudhury, Pavan K. Kancharla, Neelakantan Arumugam

**Affiliations:** Department of Biotechnology, School of Life Sciences, Pondicherry University, Pondicherry, India

**Keywords:** *FAD2* gene, fatty acid desaturase, fatty acids, cold and salt stress, plant development

## Abstract

Vegetable oils rich in oleic acid are more desirable than oils rich in polyunsaturated and saturated fatty acids. The biological switch of oleic acid to linoleic acid is facilitated by fatty acid desaturase 2 enzyme that is further classified into FAD2-1, FAD2-2, FAD2-3, and FAD2-4. The genes coding these enzymes have high sequence similarity, but differ mostly in their expression patterns. The seed-type *FAD2* genes had evolved independently after segregation by duplication from constitutively expressed *FAD2* genes. Temperature, light and wounding effectively regulate *FAD2* expression in plants. *FAD2* genes are expressed differently in different tissues of the plant, and the over-expression of *FAD2* modifies physiological and vegetative characteristics. The activity of FAD2 leads to an increase in the content of dienoic fatty acids, and hence increases the resistance toward cold and salt stress. The thorough study of the *FAD2* gene is important for understanding the expression, regulation and mechanism that will help in improving the quality of oil and stress resistance in plants.

## Introduction

Vegetable oils are important renewable resources rich in fatty acids that are broadly used in industrial applications and as an important supplement in the human diet (Yang and Xu, 2007). Fatty acids and their derivatives are not only energy reserves in plant seeds, but also play key roles in plant metabolism, membrane structural components, and signaling molecule precursors that are involved in stress-response and plant development (Ohlrogge and Browse, 1995; Harwood, 1996; Weber, 2002). Fatty acids are synthesized from acetyl-CoA in the plastids, later exported into the cytosol, and finally oil is synthesized in the endoplasmic reticulum (ER) (Browse and Somerville, 1991). The 30 enzymatic reactions taking place in the stroma of plastids produce C16- and C18-carbon fatty acids, about of which 75% is unsaturated (Ohlrogge and Browse, 1995; Somerville et al., 2000). Desaturation of the membrane phospholipids takes place by desaturases of the membrane-bound ER and chloroplast. The desaturases 2 and 3 (FAD2 and FAD3) that are integral membrane proteins in the ER primarily desaturate extra chloroplast lipids (Los and Murata, 1998; Shanklin and Cahoon, 1998). The desaturation of stearic acid (C18:0) to oleic acid (C18:1) is catalyzed by stearoyl-acyl carrier protein desaturase (SAD). Further desaturation of oleic acid to linoleic acid (18:2) is catalyzed by FAD2 in the ER and FAD6 in the plastid, whereas linoleic acid desaturation to γ-linolenic acid (C18:3, n6) is catalyzed by FAD3 in the ER and FAD7/FAD8 in the plastid (Zhang et al., 2012; Bhunia et al., 2016).

Linoleic and linolenic acids are polyunsaturated fatty acids (PUFAs) that are essential for health and nutrition, as these cannot be synthesized in humans and have to be supplied through diet (Guan et al., 2012b). Despite health benefits of PUFAs, they make the edible oil more vulnerable to rancidity, decrease its flavor, and shorten its shelf life (Pandey et al., 2014). The oxidative stability and nutritional value of the edible oil are dependent upon the fatty acid content of the oil, especially of oleic and linoleic acids (Cao et al., 2013). Oleic acid was found to have higher oxidative stability than linoleic acid, resulting in the extension of its shelf life (Ge et al., 2015). Therefore, there is a high demand for premium quality oil rich in monounsaturated fatty acids and poor in PUFAs. Such oils are more desirable, both nutritionally and commercially (Khadake et al., 2009; Guan et al., 2012b). Consumption of oils rich in monounsaturated fatty acids helps to reduce cholesterol, suppresses tumor formation, and protects from inflammatory diseases (O’Byrne et al., 1997; Yamaki et al., 2005). Therefore, increasing the oleic acid content in the oil is important for the development of oilseed crops to produce stable and healthy oils (Ge et al., 2015). The desaturation of fatty acids is one of the important biochemical processes that define the quality and economic significance of the vegetable oil (Guan et al., 2012b).

In this review, we will discuss about the features and scope of the *FAD2* gene. We shall also focus on the regulation, characterization and expression of the gene, and review the role of the gene in fatty acid biosynthesis, plant development, cold and salt tolerance, and also the future prospects in altering the gene for improvement of oilseed crops, and hence the quality of the oil.

## Fatty Acid Desaturase Genes

**FIGURE 1:**
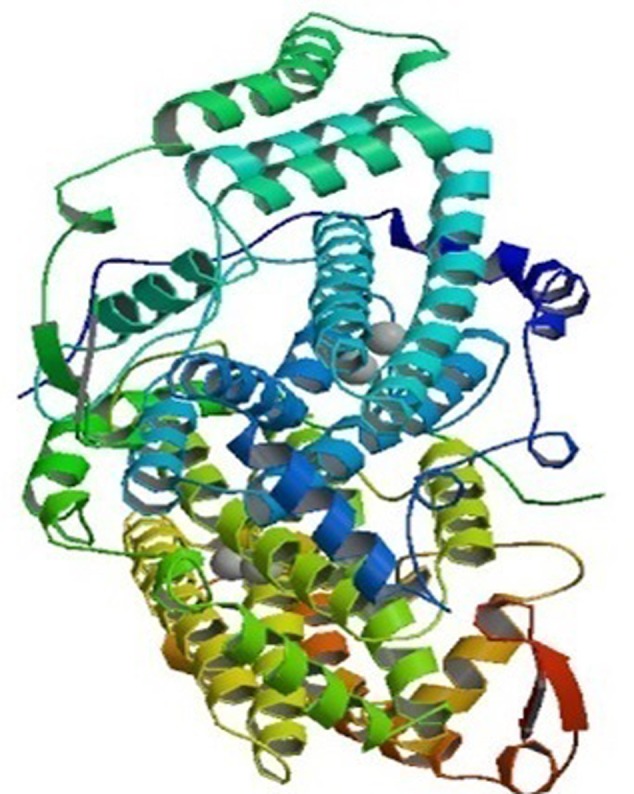
The crystal structure of stearoyl acyl protein desaturase of castor bean. The 2.40 A resolution structure shows two metal ions representing Fe-II (spheres) located at the center of the 4-helical bundle. Picture obtained from the RCSB PDB with ID 1AFR (Source: www.rcsb.org) (Lindqvist et al., 1996).

Plants have numerous fatty acid desaturase enzymes, which desaturate the majority of glycerolipids present in the tissues. The desaturases are mainly soluble or membrane bound, and present in chloroplasts and the ER, respectively. These enzymes are divided into three major classes: acyl-CoA, acyl-lipid and acyl-ACP desaturases. The acyl-CoA membrane-bound desaturases associated with ER are normally found in animals, yeast and fungi. These enzymes insert unsaturated bonds into the CoA esters of the fatty acid. These acyl-CoA desaturases, for example, the Δ5, Δ6, and Δ9 acyl-CoA are the electron acceptors of the electron-transport complex that contain cytochrome b5 and NADH-dependent cytochrome b5 reductase (Mitchell and Martin, 1995). In contrast, the desaturases in the cytoplasm of plant cells require a system that consists of cytochrome b5 and a NADH:cytochrome b5 oxidoreductase (Kearns et al., 1991). The Δ-9 desaturase synthesizes oleic acid that is used for phospholipid and cholesteryl ester synthesis. Delta-6 and Δ5 desaturases are required for the synthesis of highly unsaturated fatty acids (HUFAs), which are mainly esterified into phospholipids, and helps in maintaining membrane fluidity. The role of HUFAs may be for cold tolerance in plants and fish, and cell signaling in mammals (Nakamura and Nara, 2004). The other class of integral membrane-bound desaturases is the acyl-lipid desaturases found in cyanobacterial cells and chloroplasts. They introduce double bond into the fatty acyl chain of polar glycerolipids and use ferredoxin as the electron donor (Los and Murata, 1998; Tocher et al., 1998). The structure of this enzyme is similar to acyl-CoA desaturases, which are mainly transmembrane proteins (Murata and Wada, 1995). The Δ3, Δ6, Δ9, and Δ12-acyl-lipid desaturases have been characterized and are specified by the double bond that is inserted nearest the carboxyl or the methyl terminus of the fatty acid (Reddy et al., 1993; Sakamoto et al., 1994; Murata and Wada, 1995; Sakamoto and Bryant, 1997). The soluble desaturases, for instance, acyl-ACP desaturases, present in the plastidial stroma of plants, utilize ferredoxin as the electron donor. They incorporate double bonds in fatty acids that are esterified with acyl carrier protein (ACP). The best example is the Δ9 acyl-ACP desaturase, which catalyzes desaturation of stearic to oleic acid in the stroma of chloroplasts. This desaturase enzyme predominantly converts saturated fatty acids to unsaturated ones in vegetable oils (Slabas and Fawcett, 1992). The crystallographic examination of stearoyl-ACP desaturase isolated from castor seeds (*Ricinus communis*) indicates that the desaturase forms a di-iron-oxo active center with the two iron atoms bound in the symmetric structure (**Figure 1**). The two iron atoms interact with O_2_ with a consensus-binding motif of [(D/E)X2H2] (Fox et al., 1993; Los and Murata, 1998; Shanklin and Cahoon, 1998). The deep channel of an extended surface may be the fatty acyl chain binding site and the substrate, stearic acid in this channel places the Δ9-carbon atom in the neighborhood of one of its iron ion (Lindqvist et al., 1996).

Membrane-bound desaturases, such as the Δ12- and Δ15-desaturases convert oleate into linoleate and linolenate, respectively. Since these desaturases are ER integral membrane proteins and are quite difficult to isolate and characterize by biochemical methods. The information about plant desaturases has been obtained by isolation and characterization of a series of mutants involved in lipid biosynthesis in *Arabidopsis thaliana.* There are four chloroplast desaturase loci: *FADA, FADB, FADC* and *FADD*, which are also called as *FAD4, FAD5, FAD6*, and *FAD7*, respectively (Ohlrogge and Browse, 1995). The microsomal oleate Δ12-desaturase or fatty acid desaturase 2 (FAD2; EC 1.3.1.35) is a hydrophobic transmembrane ER protein, acting on fatty acids, inserting a *cis* double bond between the C12 and C13 position of monounsaturated oleic acid, thereby producing polyunsaturated linoleic acid (Ohlrogge and Browse, 1995; Shanklin and Cahoon, 1998; Somerville et al., 2000). FAD2 is the key enzyme accountable for biosynthesis of polyunsaturated fats in non-photosynthetic tissues, for instance, roots and developing seeds of oilseed plants (Miquel and Browse, 1992; Zhang et al., 2012). The FAD2 in the ER utilizes phospholipids as substrates with NADH, NADH-cytochrome b5 reductase, and cytochrome b5 as electron donors. On the other hand, the plastidial oleate desaturase (FAD6) primarily uses glycolipids as acyl carriers, and ferredoxin reduced by ferredoxin-NAD(P) reductase as electron donors (Hernández et al., 2009). The *FAD2* gene was first reported in Arabidopsis with a single copy, which is constitutively expressed (Okuley et al., 1994; Beisson et al., 2003). Further studies have identified more than one *FAD2* gene in a variety of crops, such as sesame (*Sesamum indicum*), corn (*Zea mays*), canola (*Brassica napus*), olive (*Olea europaea*), soybean (*Glycine max*), sunflower (*Helianthus annuus*), and cotton (*Gossypium hirsutum*) (Jin et al., 2001; Pirtle et al., 2001; Kinney et al., 2002; Hernández et al., 2005; Li et al., 2007; Rolletschek et al., 2007; Kargiotidou et al., 2008). *FAD2* is 1,164 bp long with an open reading frame coding for about 387 amino acids. The *FAD2* gene consists of a single large intron in the 5′-untranslated region (UTR), which is evolutionarily conserved. However, the exon number may vary across the plant species, for example, Arabidopsis, castor bean, and soybean had only one exon, in contrast, Indian mustard contains two (Okuley et al., 1994; Chi et al., 2011a; Sharma and Chauhan, 2012; Suresha and Santha, 2013). The intron could be important for transcriptional regulation of *FAD2* gene expression. The *FAD2* gene has been classified into four types, namely, *FAD2-1, FAD2-2, FAD2-3*, and *FAD2-4* on the basis of their site and pattern of expression. The four variations of the *FAD2* gene show high sequence similarity, but show differences in their expression patterns and functions in fatty acid modification (Kongcharoensuntorn, 2001). The FAD2-1 is a seed-specific desaturase that synthesizes polyunsaturated fatty acids in young seed and developing flower buds (Liu et al., 2001). *FAD2-2* is expressed at a low level from vegetative stage to maturing phase during seed development (Pirtle et al., 2001). *FAD2-2* is the major gene responsible for the synthesis of linoleic acid (Hernández et al., 2009). FAD2-3 and FAD2-4 synthesize mostly polyunsaturated fatty acids almost in all the tissues. It was reported that FAD2-4 has 98% similarity with the FAD2-3 polypeptide (Zhang D. et al., 2009). Similarly, FAD6 is also ω-6 desaturase, synthesizing linoleic acid from oleic acid in plastids, unlike FAD2 in ER. The ω-3 desaturases, such as FAD3, FAD7 and FAD8 synthesize linolenic acid (C18:3) from linoleic in the ER (FAD3) and plastids (FAD7 and FAD8) (Gibson et al., 1994; Berberich et al., 1998). The FAD3 is a microsomal enzyme located in the ER facing the cytosol, whereas FAD7 and FAD8 are plastidial enzymes located in the inner membrane of the chloroplast envelope. In addition, FAD7 can also be found in the thylakoid of the chloroplasts (Andreu et al., 2007; Bhunia et al., 2016). In contrast to this, FAD4 and FAD5 produce C16:1 from C16:0 in particular for phosphatidyl glycerol and mono-galactosyldiacylglycerols (MGDG), respectively (Murphy and Piffanelli, 1998).

**FIGURE 2:**
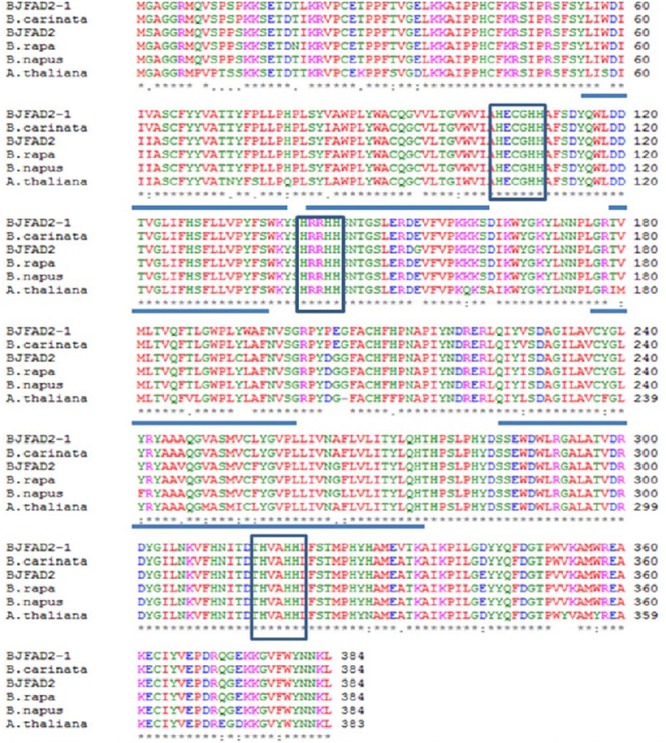
Comparative amino acid sequence alignment of FAD2 genes in *Brassica juncea(X91139), B. rapa* (AJ459107), *B. napus* (AF243045), *B. carinata* (AF124360) and *Arabidopsis thaliana* (L26296). Boxes represent three histidine motifs (HXXXHH, HXXHH and HXXHH) and underlined regions represent five hydrophobic regions (Image taken from Suresha et al., 2012) (This picture is reproduced here after taking permission from copyright holder).

About 20 distinct motifs were identified in fatty acid desaturases. These motifs mainly belong to the transmembrane region of unknown complexity. Proteins of the FAD2 subfamily contain the motifs 2, 5, 6, 15, 16, and 17. The FAD2 enzyme contains 6 transmembrane domains and 8 conserved histidine residues in three clusters (HXXXH, HXXHH, and HXXHH), harboring eight iron-binding domains necessary for reduction of oxygen during desaturation, and is characteristic of all membrane-bound desaturases (Okuley et al., 1994; Shanklin et al., 1994) (**Figure 2**). It was found that four (valine, alanine, leucine, valine) out of the eight amino acids (threonine, histidine, valine, alanine, histidine, histidine, leucine, valine) belonged to hydrophobic residues in the third histidine cluster, which implied that these hydrophobic residues may be located in the interior of the active site of the enzyme. The top ten amino acids were all found to be leucine in the second histidine cluster (Tanhuanpaa et al., 1995; Ge et al., 2015). This structure might be one of the active sites of the FAD2 enzyme. Moreover, threonine was identified as the last three residues in the second histidine cluster and as the last four residues in the third histidine cluster, which play an important key role in the desaturation and hydroxylation of the FAD2 enzyme (Broadwater et al., 2002; Ge et al., 2015). The histidine boxes contain the catalytic center, which forms ligands to a diiron cluster, and exchange of a histidine with a different amino acid disrupts the function of desaturase (Shanklin et al., 1994; Shanklin and Cahoon, 1998; Guan et al., 2012b). The amino acid sequence of FAD2 is almost similar in most plants (Tao et al., 2006). Bioinformatics tools didn’t show any N-terminal signal peptide for probable localization of the FAD2 protein in organelles, like Golgi body, chloroplasts, and mitochondria. The C-terminal signaling motif (YKNK) allows the FAD2 protein to bind selectively to and integrate into the ER (Nielsen et al., 1997; Schell, 1998; McCartney et al., 2004; Nayeri and Yarizade, 2014).

As we have studied different types and subtypes of *FAD* genes in plants, it would be now interesting to know the evolutionary relationship of these genes in different crops. This would make us understand, how much they are diverged from other *FAD* genes that may help us in identifying their origin and existence.

## Phylogenetic or Evolutionary Relationship of *FAD2* Genes

**FIGURE 3:**
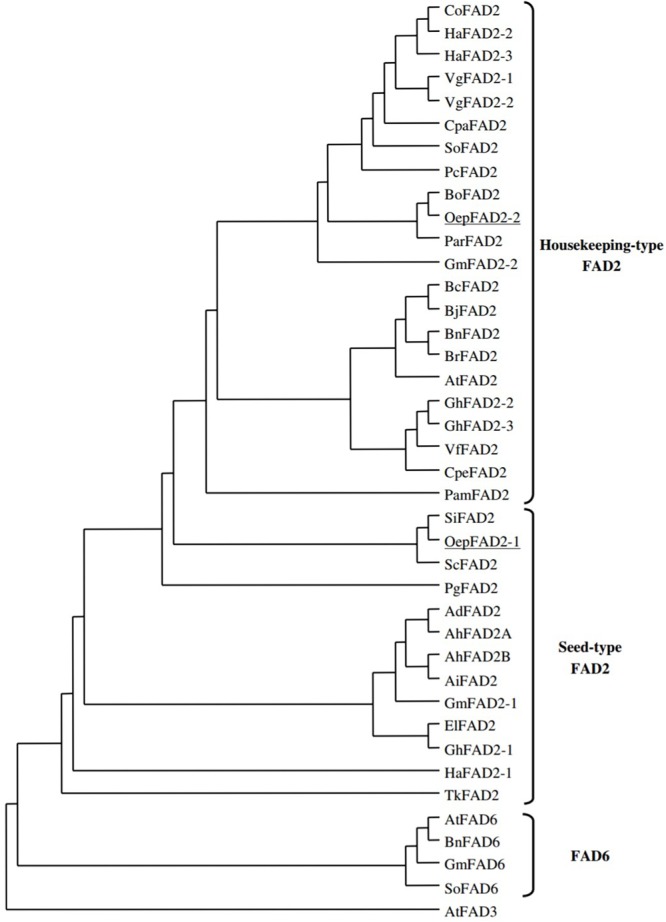
Phylogenetic analysis of FAD2 and FAD 6 enzymes. Accession numbers of the different desaturases included in the analysis are *Arabidopsis thaliana* (AtFAD2. L26296; AtFAD3_;_ D17579; AtFAD6, U*09503), Arachis duranensis* (AdFAD2_:_ AF272951), *Arachis hypogaea* (AhFAD2A_,_ AF030319; AhFAD2B, AF272950), *Arachis ipaensis* (AiFAD2_:_ AF272952), *Borago officinalis* (BoFAD2_:_ AF074324), *Brassica carinata (BcFAD2_:_* AF124360), *Brassica juncea* (BjFAD2_:_ X91139), *Brassica napus* (BnFAD2_;_ AF243045; BnFAD6_:_ L29214), *Brassica rapa* (BrFAD2_:_ AJ459107), *Calendula officinalis* (CoFAD2_:_ AF343065), *Crepis palestina* (CpaFAD2_;_ Y16284), *Cucurbita pepo* (CpeFAD2_:_ AY525163), *Euphorbia lagascae* (E1FAD2, AY486148), *Glycine max* (GmFAD2-l_,_ L43920; GmFAD2-2_,_ L43921; GmFAD6, L29215), *Gossypium hirsutum* (GhFAD2-l_,_ X97016; GhFAD2-2_,_ Y10112; GhFAD2-3_,_ AF331163), *Helianthus annuus* (HaFAD2-1, AF251842; HaFAD2-2, AF251843; H3FAD2-3, AF251844), *Persea americana* (PamFAD2_,_ AY057406), *Petroselinum crispum* (PcFAD2_:_ U86072), *Punica granatum* (PgFAD2_,_ AJ437139), *Sesamum indicum* (SiFAD2, AF192486), *Solanum commersonii* (SoFAD2_;_ X92847), *Spinacia oleracea* (SoFAD2_;_ AB094415; SoFAD6_;_ X78311), *Trichosanthes kirilowii* (TkFAD2; AY188445), *Vernicia fordii* (VfFAD2_,_ AF525535), *Vernonia galamensis* (VgFAD2-1, AF188263; VgFAD2-2_,_ AF188264) (Picture taken from Hernández et al., 2005) (This picture is reproduced here after taking permission from copyright holder).

The phylogenetic relationships of *FAD2* genes were explained clearly by alignment of their coded amino acid sequences with other *FAD* genes of oil seed crops. A dendrogram was constructed that included all plant oleate desaturase enzymes, either plastidial (FAD6) or microsomal (FAD2). As shown in **Figure 3**, the coding sequences of ω-6 fatty acid desaturase in plants were classified into three main groups, namely, house-keeping *FAD2*, seed type *FAD2* and *FAD6* (Hernández et al., 2005; Guan et al., 2012a,b; Suresha et al., 2012). The huge separation between branches of *FAD2* and *FAD6* showed that they had diverged during early gene evolution (Reiser et al., 2000). The *FAD2* seed-type genes have evolved independently after segregation by duplication from constitutive expressed *FAD2* genes (Martínez-Rivas et al., 2001). This also suggests that the ancestral *FAD2* gene had diverged prior to speciation, and the diverged *FAD2* genes with the same function were probably found to be either seed-type or constitutive (Lee et al., 2012).

## Regulation of *FAD2* Gene

**FIGURE 4:**
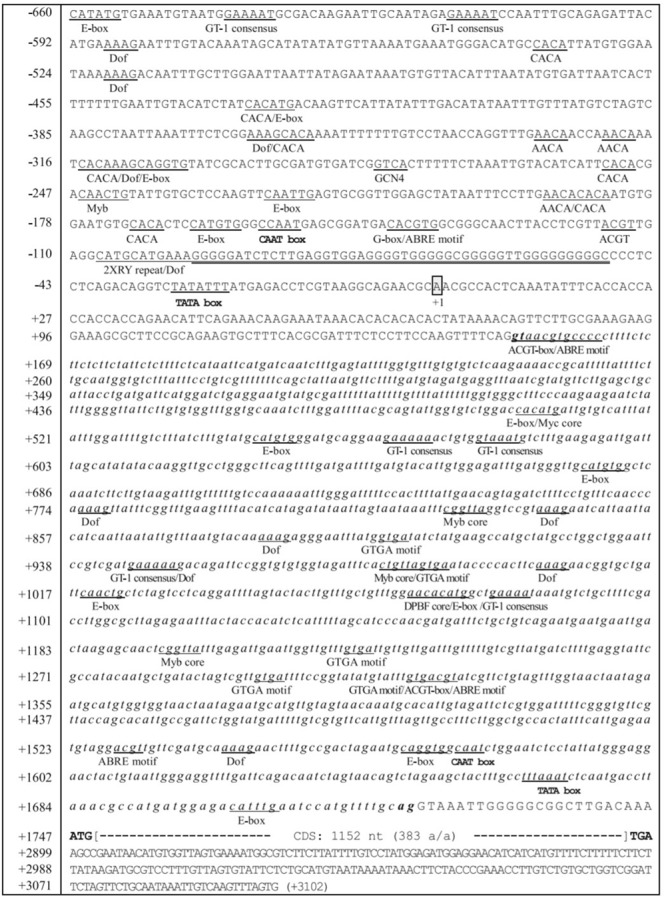
Nucleotide sequences of the 5′-flanking region, intron and regulatory elements of the *Sesamum* FAD2 gene. A box represents the transcription initiation site designated by the + 1 position. The numbering on the left refers to the nucleotide sequences. The sequences of the *SeFAD2* intron are shown in small italic letters. The putative TATA box occurs between -25 and -31. Translational initiation (ATG) and termination codon (TGA) are in bold, and the coding region of the *SeFAD2* gene (1,152 nucleotides; 383 amino acids) is shown by large brackets. Several potential regulatory *cis*-elements are underlined and designated with the names of each of the motifs. G-rich sequences are double-underlined with thick appearance between -49 and -96 (Image taken from Kim et al., 2006) (This picture is reproduced here after taking permission from copyright holder).

It would be interesting to look out on, how these divergent *FAD* genes regulate fatty acid desaturation in plants and carry out the specific functions. The transcriptional control of the *FAD* genes occurs through the promoter DNA and promoter DNA-specific regulatory proteins. The regulation has been clearly explained in sesame by Kim et al. (2006). The transcriptional control of the *FAD2* gene during seed development is spatially and temporally regulated. The region from -179 to -53 in the sesame *FAD2* promoter contained positive *cis*-elements for *FAD2* gene expression. On the other side, the *FAD2* promoter region from -547 to -180 region harbors negative *cis*-elements for the gene repression. The eight potential *cis*-elements in developing seeds that regulate the gene expression are (CA)2 element, E-box (CANNTG), CCAAT box, ABRE motif (ACGTGKC), G-box (CACGTG), G-box-like element (ACGT), Prolamin-box (AAAG), and RY repeat element (CATGCA) (**Figure 4**). The *SeFAD2* gene expression revealed that abscisic acid (ABA) was responsible for the regulation during seed development. In the *SeFAD2* promoter, ABA-responsive elements were found from the region of -660 to -548 and -179 to -53 (Kim et al., 2006). The *FAD7* promoter contains *cis*-acting elements, such as BoxII (GT-1 sites) and G-box-like (CCACTTGG) motifs that are members of light-responsive promoters (Guilfoyle, 1997). Different *cis*-regulatory elements in *FAD2* promoter are involved in the abiotic and biotic stress responses, and influence the control of gene expression, specifically in seeds (Nayeri and Yarizade, 2014).

It was also reported that the regulatory mechanism based on introns was also involved in the expression of plant *FAD2* genes (Kim et al., 2006). Xiao et al. (2014) reported that both promoter and intron are involved in controlling the expression of *FAD2* gene in *Brassica napus*. The quantitative trait locus (QTL) analysis and genome-wide association studies (GWAS) using the multiparent advanced generation intercross (MAGIC) population in Arabidopsis, suggested that ω-6 desaturation can be largely controlled by *cis*-acting sequence variants of the *FAD2* intron (Menard et al., 2017).

The regulation of *FAD2* gene may be important in understanding the fatty acid composition of plant membranes and membrane fluidity in the cold tolerance of plants (Kongcharoensuntorn, 2001). Fatty acid desaturase genes in plants normally undergo different types of regulation by temperature, light, and wounding.

### Temperature

Temperature is a major environmental factor that regulates fatty acid desaturation in plants. The regulation of gene expression appears to vary with the species, tissue, and gene. The effect of temperature on the *FAD* gene expression levels was well studied in olive fruit (Hernández et al., 2009, 2011). At 15°C, a small increase of *FAD6* transcript levels was observed with a temporary induction of *FAD2* genes (slight for *FAD2-1* and intense for *FAD2-2*). But when the temperature was increased from 15°C to 35°C, the expression of *FAD2* genes was decreased. Similarly, an upregulation of *FAD2* gene expression by cold stress was observed in avocado fruits and cotton cotyledons (Wang et al., 2004; Kargiotidou et al., 2008; Teixeira et al., 2009). On the contrary, when Arabidopsis and soybean cultures were grown under low temperatures, no such significant differences were observed in expression levels of *FAD2* and *FAD6* genes (Okuley et al., 1994; Heppard et al., 1996). Low temperature normally increases the content of polyunsaturated fatty acids that maintain the fluidity of membranes in cold climate (Los and Murata, 1998; Routaboul et al., 2000). The *fad6* mutant of Arabidopsis had a high level of monounsaturated fatty acids and reduced levels of polyunsaturated fatty acids in membrane lipids, and mutants, such as *fad5, fad6*, and *fad3: fad7: fad8* were found more vulnerable to photoinhibition as compared to wild-type plants after chilling treatment (Zhang et al., 2012). Recently, Botella et al. (2015) depicted the role of ALA10, a P4 type-ATPase that interacts with *FAD2* gene and affects fatty acyl desaturation by downregulating the *FAD2* and *FAD3* activities in Arabidopsis leaves. The effect of ALA10 on leaf development was found significant, when plants were grown at chilling temperatures (10°C). These studies showed a significant up-regulation of *FAD2* or *FAD3* isogenes at a lower temperature, whereas some studies have also observed either down-regulation or no appreciable change.

The change in gene expression levels and protein content with temperature was observed in case of Δ9, Δ12, and Δ15 desaturases (Vega et al., 2004; Teixeira et al., 2009, 2010). It was reported that two domains in FAD2-1 were important in mediating temperature-dependent stability of FAD2-1A isoform. In addition, the N-terminus of FAD2-1 and FAD3 was shown to be important in the temperature-dependent turnover of proteins. The FAD2 and FAD3 proteins were thus less stable and hence, less abundant at high temperatures (Khuu et al., 2011). Domain-swapping and mutagenesis experiments revealed that each protein contained a degradation signal in its N-terminus, and the PEST-like sequence within this region was largely responsible for the rates of protein turnover. A PEST is a signal sequence present on a protein that confers rapid protein degradation at high temperatures, usually via the ubiquitination/26S proteasome (UPS) pathway. The E3 ligases seem to play an important role in determining the substrate specificity of proteins degraded through this pathway (Elsasser and Finley, 2005). In addition, the protein degradation requires other specific components of the ER including the Cdc48 adaptor proteins Doa1, Shp1, and Ufd2. It was thus clearly indicated that FAD2 and FAD3 protein abundance was regulated by a combination of *cis*-acting degradation signals and the UPS pathway, and the modulation of these protein amounts in response to temperature may represent one mechanism of homeoviscous adaptation in plants (Schrader et al., 2009; O’Quin et al., 2010).

The other way of regulating desaturase activity was by post-transcriptional mechanisms as observed in wheat roots for *FAD3, FAD8* in Arabidopsis leaves, and seed-specific *FAD2-1* in soybean (Horiguchi et al., 2000; Matsuda et al., 2005; Tang et al., 2005). Li et al. (2007) found that FAD2-3 increased di-unsaturated fatty acids under cold stress due to post-transcriptional/post-translational modifications of gene rather than increase in mRNA levels. Promoter analysis of *FAD-2* gene showed that the response of the gene under low temperatures could be due to the interaction of various factors generating signaling pathways, which would then determine gene response and plant adaptation to the new condition. The activation of the acclimation genes induced by ABA takes place by ABA-dependent and ABA-independent pathways. The promoter analysis of the *FAD2* from *G. hirsutum* and *A. thaliana* indicated that ABRE1/2 and ABRE3 elements were present in the promoters of *FAD2-3* and *FAD2-4* of *G. hirsutum*, but absent in the promoters of *FAD2* from *A. thaliana* (Kargiotidou et al., 2008).

The glycerolipid pathway in Arabidopsis was also involved under temperature stress and was largely acting by rebalancing of the two pathways. In *Arabidopsis thaliana*, the prokaryotic (chloroplast) pathway was upregulated in response to low temperature that induced MGDG biosynthesis, whereas high temperature had increased di-galactosyldiacylglycerols (DGDG) biosynthesis by redirection of acyl channeling through the eukaryotic pathway (ER) (Li et al., 2015). It was reported that serine-185 of FAD2-1 enzyme was phosphorylated by calcium-dependent protein kinases (CDPKs) during seed development. The serine motif was found conserved in the amino acid sequence of many plant FAD2 enzymes. The expression studies showed that phosphorylation had downregulated enzyme activity, but there was no connection found between serine-185 phosphorylation and temperature regulation (Huang and Huber, 2001; Tang et al., 2005).

### Light

Light is another factor that affects desaturation of fatty acid in plants. A light-dependent increase of polyunsaturated fatty acids was reported in photosynthetic tissues. There was an increase in linoleic and α-linolenic acids in cotyledons of cucumber in response to light (Murphy and Stumpf, 1979), however, light had elevated only the α-linolenic acid level in oat leaves and Arabidopsis callus (Ohnishi and Yamada, 1983; Brockman et al., 1990). To confirm the effect of light on fatty acid desaturation, soybean cell cultures and olive fruits were kept under darkness, such effect decreased *FAD3* and *FAD8* expression levels in former and decreased *FAD2* expression levels in latter, hence indicating a light-dependent transcriptional regulation of *FAD* genes (Collados et al., 2006; Hernández et al., 2011). It was also reported that the expression of *FAD2-3* and *FAD2-4* genes under cold stress was found light-dependent and was because of an indirect hormonal effect or a direct effect of light regulatory elements on the *FAD2-3* and *FAD2-4* promoters. It is known that light inhibits ethylene synthesis, which in turn affects auxin distribution. Thus the influence of light on gene expression under cold stress could be regulated by the ethylene/auxin gradient, which in turn, is regulated by light (Vandenbussche et al., 2003; Kargiotidou et al., 2008).

### Wounding

Plants use linoleic and α-linolenic acids as signaling precursor molecules for defense system against pathogen attack and wounding (Farmer, 1994). The *FAD7* gene induced by wounding with the parallel increase of α-linolenic acid was reported in many plants (Teixeira et al., 2010). Further, the increase of FAD2 and Δ9 stearoyl-ACP desaturase levels was also reported in avocado fruits when infected with *Colletotrichum* (Wang et al., 2004), and the induction of parsley *FAD2* and *FAD7* genes by a fungal elicitor (Kirsch et al., 1997a,b). The FAD2 enzyme was thought to be involved in the wounding reaction by increasing the biosynthesis of linoleic and palmito-linoleic acid in the ER of olive fruit. It was reported that F*AD2-1* and *FAD2-2* expression levels were slightly increased on the injury, whereas *FAD6* transcript levels were not altered in response to wounding. In addition, the presence of palmito-linoleic acid was also noticed in microsomal lipids, but not in plastids after wounding (Hernández et al., 2011). Such reports clearly indicate that *FAD* genes are also regulated by wounding and pathogen attack.

## *FAD* Genes Isolation and Characterization

The research on *FAD* genes has been progressed well from the past to present. Here in this column, we will give some idea about isolation and characterization of *FAD* genes, especially *FAD2* gene from the year 1990 to 2016 (**Table 1**). The techniques often used for conducting such study were reverse transcription-polymerase chain reaction (RT-PCR), rapid amplification of cDNA ends (RACE), and real-time quantitative PCR (RT-qPCR). The *FAD2* gene was first cloned and identified in Arabidopsis by the T-DNA tagging method (Okuley et al., 1994). Hernández et al. (2009) studied the correlation between the expression level of *FAD2* and *FAD6* genes with the linoleic acid level in olive fruit. They observed an increase of linoleic acid with the increase in *FAD2-2* gene expression level, and thus concluded that *FAD2-2* is the main gene responsible for the linoleic acid content. To elucidate the connection of Δ12 *FAD* in salt and freezing tolerance, Lu et al. (2010) identified the role of *FAD6* gene in low temperature and high salinity acclimatization in Antarctic microalga, *Chlorella vulgaris* NJ-7. The three *FAD2* genes that were isolated and characterized in *Camelina sativa* were tissue and developmentally regulated (Kang et al., 2011). Chi et al. (2011a) identified orthologous genes from soybean encoding fatty acid desaturases. The identification of these desaturases is useful for the reconstruction of the pathways concerned in the biosynthesis of unsaturated fatty acids. Zhang et al. (2011) cloned and characterized cDNAs encoding fatty acid desaturases from lima beans, and their expression patterns were investigated in different tissues under various stress conditions, especially low temperature. Their results provided a better understanding of regulation and structure of the desaturase genes in plants. Guan et al. (2012c) used microarray technology for determining the distinct gene clusters linked with the oleic acid synthesis in rapeseed. The comparison of gene expression profiles between high and low-oleic acid genotypes grouped a large number of differentially expressed genes linked with the high-oleic acid trait. This provided a basis for further studies on the mechanisms behind oleic acid synthesis and accumulation. Lee et al. (2012) reported the cloning and characterization of two distinct *FAD2* genes from *Vitis labrusca*, and revealed their differential expression patterns. The *FAD2* genes from *V. labrusca* functionally complemented the *FAD2* mutation in Arabidopsis. Suresha et al. (2012) isolated *FAD2* gene through RT-PCR technique from *Brassica juncea*, and studied its tissue- and growth temperature-dependent expression. They observed that *FAD2* gene is developmentally regulated with increased expression in mid-maturation stage as compared to early and late stages of seed development. The results also showed one-fold higher and three fold lower expression rate of *fad2* under the lower and the higher temperature, respectively. Cao et al. (2013) isolated and characterized a *FAD2* gene family from safflower by RT-qPCR. The phylogenetic analysis reported their non-allelic nature and was evolved by gene duplication. The functional divergence of the *FAD2* family was discovered by heterologous and transient expression in yeast and *Nicotiana benthamiana*, respectively. Chen et al. (2015a) cloned *FAD2* from *Vernicia fordii* and transformed in *Rhodotorula glutinis*, and observed the synergistic effect on unsaturated fatty acid metabolism. Recently, Sun et al. (2016) cloned and identified the role of microsomal *FAD2* from *Elaeis guineensis*. They reported that FAD2 used only oleic acid as a substrate and converted it to linoleic acid. The characterization of the above *FAD* genes will help the researchers in the construction of the pathways involved in biosynthesis of unsaturated fatty acids, and can provide many candidate genes for the genetic engineering of stress tolerance in plants.

**Table 1:** Characterization of *FAD* genes from different plant sources.

S. no	Gene type	Technique used	Plant source	Reference
1	Plastidial ω-6 desaturase	RACE	*Spinacia oleracea*	Schmidt and Heinz, 1990
2	*FAD3*	Map based cloning	*Arabidopsis thaliana*	Arondel et al., 1992
3		T-DNA tagging	*Arabidopsis thaliana*	Yadav et al., 1993
4		Chromosome mapping	*Oryza sativa* L.	Kodama et al., 1997
5	*FAD7*	Chromosome walking	*Arabidopsis thaliana*	Iba et al., 1993
6	*FAD8*	Heterologous hybridization	*Arabidopsis thaliana*	Gibson et al., 1994
7	*FAD2*	T-DNA tagging	*Arabidopsis thaliana*	Okuley et al., 1994
8			*Olea europaea* cv. Picual	Hernández et al., 2005
9		PCR	*Linum usitatissimum L.*	Khadake et al., 2009
10		RT-PCR	*Zea mays*	Tao et al., 2006
11			*Davidia involucrata*	Lei et al., 2010
12			*Camelina sativa* L.	Kang et al., 2011
13		RT-qPCR and RACE	*Carthamus tinctorius* L	Guan et al., 2012a
14			*Elaeis guineensis*	Sun et al., 2016
15		Southern blot and Semi-quantitative Real-time PCR	*Vitis labrusca*	Lee et al., 2012
16		RT-qPCR	*Brassica juncea*	Suresha et al., 2012
17			*Carthamus tinctorius* L.	Cao et al., 2013
18			*Vernicia fordii*	Chen et al., 2015a
19		Colony PCR and gene sequencing	*Brassica juncea*	Suresha and Santha, 2013
20	ω-6 fatty acid desaturase gene	using novel probes derived from amino acid conserved sequences	*Glycine max* and *Brassica napus*	Hitz et al., 1994
21	*FAD2-2*	RT-qPCR	*Olea europaea* L.	Hernández et al., 2009
22	*FAD2-4*	RT-PCR	*Gossypium hirsutum* L.	Zhang D. et al., 2009
23	*FAD6*	RT-PCR and RACE	*Chlorella vulgaris* NJ-7	Lu et al., 2010
24	*FAB2, FAD2, FAD3, FAD5, FAD6, FAD7, FAD8, SLD1* and *DES1*	Gene annotation	*Glycine max*	Chi et al., 2011a
25	*FAB2, FAD2-2, FAD6* and *SLD1*	RT-qPCR	*Arachis hypogaea* L	Chi et al., 2011b
26	*SAD, FAD2* and *FAD3*	RACE	*Phaseolus lunatus* L.	Zhang et al., 2011
27	Gene clusters linked with the oleic acid synthesis	Microarray technology	*Brassica napus*	Guan et al., 2012c
28	*FAD2-1, FAD2-2*, and *FAD2-3.1*	RT-PCR	*Camelina sativa* L.	Kim et al., 2014
29	*SAD1, SAD2, FAD2, FAD2-2, FAD3A* and *FAD3B*	Real-time PCR	*Linum usitatissimum* L.	Rajwade et al., 2014
30	*FAD2-1, FAD2-2, FAD2-3, FAD2-4, FAD2-5*, and *FAD2-6*	RT-qPCR	*Arachis hypogaea* L.	Wang et al., 2015
31	5′-flanking region of the *FAD2-1* gene	hiTAIL-PCR and RT-PCR	*Gossypium hirsutum*	Liu et al., 2015

*RACE, rapid amplification of cDNA ends; RT-PCR, reverse transcription-polymerase chain reaction; RT-qPCR, real-time quantitative PCR; *FAD*, fatty acid desaturase; *SAD*, stearoyl-acylcarrier protein desaturase; *SLD*, sphingolipid Δ8 desaturase; *DES*, sphingolipid Δ4 desaturase; *FAB*, stearoyl-ACP desaturase; hiTAIL-PCR, high-efficiency thermal asymmetric interlaced PCR.*

Genetic engineering is a recent useful technique of crop improvement that produces novel plant varieties. The genetic engineering with the aim of genetic improvement was achieved by silencing the *FAD2* gene to increase oleic acid and simultaneously to reduce linoleic acid. This was reported in various plants, such as canola (Stoutjesdijk et al., 2000), cotton (Liu et al., 2000), groundnut (Yin et al., 2007), Jatropa (Utomo et al., 2015), and linseed (Chen et al., 2015b). It was reported that the plastidic desaturase activities are partly accountable for the instability of the high-oleic acid *FAD2*-silenced plants (Zhang et al., 2016). Marine algae were known to possess certain functional activities that help in the lipid metabolism, and hence can be useful for human and animal nutrition. Norambuena et al. (2015) conducted such type of study that assessed the potentials of two commercially available algae for including them into diet of Atlantic salmon fish. They reported increase in PUFA content in whole body of fish that displayed the importance of bioactive carotenoids found in algae. However, the observed increase of PUFA content in fish tissues was statistically significant, but from a nutritional point of view was considered minimal for consumers.

## Expression of *FAD2* Gene

The *FAD2* gene expression has provided many new challenges and opportunities for studying the gene in the enhancement of oil quality. The *FAD2* gene is expressed throughout the plant kingdom and is identified by Northern blot and RT PCR techniques. The functions of both *FAD2-1* and *FAD2-2* in soybean were investigated by Heppard et al. (1996). They reported that the *FAD2-1* gene was expressed strongly in developing seeds, whereas *FAD2-2* gene was constitutively expressed in both developing seeds and vegetative tissues. It was reported that FAD2-2 was mainly responsible for the conversion of oleic to linoleic acid (Jin et al., 2001). The FAD2-1 in *Olea europaea* desaturates storage lipids in young seed, whereas FAD2-2 desaturates lipids in maturing seeds and mesocarp (Hernández et al., 2005). Semi-quantitative RT-PCR was used for determining the expression pattern of *FAD2* gene in maize. The expression of *FAD2* was found higher in immature embryos than leaves, stem, and roots (Tao et al., 2006). The *FAD2-4* and *FAD2-3* genes were found to be expressed in all tissues of the cotton plant, such as seeds, seedlings, roots, stems, leaves, developing flower buds, and ovule fibers. This constitutive expression pattern was found different from the *FAD2-1* gene, which was limited to flower buds and seeds. However, the expression of a *FAD2-2* gene was expressed in all other tissues, but hardly found in hypocotyls, roots, and stems (Zhang D. et al., 2009). The results of quantitative RT-PCR found that the transcript levels of *FAD2* and *FAD2-1* in *Arachis hypogaea* L. were elevated in seed than the other tissues. Alternatively, the transcript level of *FAD2-2, FAD6*, and *SLD1* (sphingolipid Δ8 desaturase) were higher in leaves. These genes had different expression patterns with different biochemical functions throughout seed development and vegetative growth (Chi et al., 2011b). Suresha et al. (2012) reported that *FAD2* was expressed constitutively in all the tissues of *Brassica juncea* and was developmentally regulated during oil biosynthesis. The expressions of *FAD2-1* and *FAD2-3-1* in *Camelina sativa* were mainly reported in flowers and seeds (Kim et al., 2014). Transcript profiling of six microsomal desaturase genes, such as *SAD1, SAD2, FAD2, FAD2-2, FAD3A* and *FAD3B* was performed by real-time PCR (Rajwade et al., 2014). *SAD2* was the most highly expressed gene found throughout all stages of seed development. The expression of *SAD1* was lower than *SAD2* and remained constant throughout all developmental stages. Analysis of *FAD2* transcript distribution in peanut found that the *FAD2-1* gene had a 70-fold higher expression rate in developing seeds than the *FAD2-2* gene, but the *FAD2-2* gene was abundantly expressed in flowers (Wang et al., 2015). Recently, Dong et al. (2016) reported that the *FAD2* gene in *Cucumis sativus* L. was expressed in all tissues, while all other *FAD* genes were found expressed mainly in the cotyledons and leaves. The story of *FAD2* gene continues also hereafter its expression in order to play a key role in fatty acid synthesis, plant development, cold and salt tolerance for the plant survival.

## Significance and Key Role of FAD2

### Fatty Acid Biosynthesis

**FIGURE 5:**
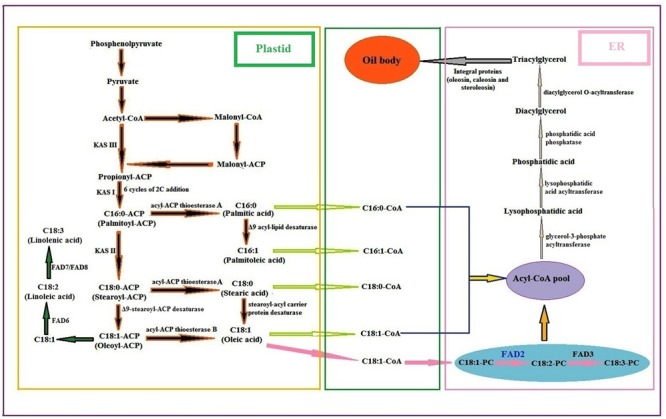
General biosynthetic pathway of fatty acids and lipids in plants [PC phosphocholine; FAD fatty acid desaturase: KAS ketoacyl-ACP synthase] (Guan et al., 2012c: Suresha et al., 2012; Bhunia et al., 2016).

The two major and dominant biosynthetic pathways, prokaryotic and the eukaryotic, work together in plant cells for the synthesis of glycerolipids and polyunsaturated fatty acids (Browse and Somerville, 1991). The biosynthetic pathway of fatty acids and lipids in plants is well presented in **Figure 5**. Many enzymes in fatty acid biosynthesis had been biochemically characterized, as well as the encoding genes from plants like *Brassica napus* and *Arabidopsis thaliana* (Slabas and Fawcett, 1992; Ohlrogge and Browse, 1995; Beisson et al., 2003; Wang et al., 2010). Fatty acids are normally synthesized by a fatty acid synthase (FAS) complex located in the plastids that uses acetyl-CoA as a precursor and malonyl-ACP as an elongator. The malonyl-thioester undergoes a series of condensation reactions with an acetyl-CoA catalyzed by a 3-ketoacyl-ACP synthase-III (KAS-III) that produces propionyl-ACP (C4:0-ACP). Subsequent condensation reactions takes place up to the formation of palmitoyl-ACP (C16:0-ACP) catalyzed by a KAS-I isoforms, and finally KAS-II elongates the C16:0-ACP to stearoyl-ACP (C18:0-ACP) (Harwood, 1996). The Δ9-stearoyl-ACP desaturase then converts most of the stearoyl-ACP to oleoyl-ACP (C18:1-ACP). Here C18:1-ACP after conversion to oleic acid (C18:1) gets desaturated to linoleic acid by the ω-6 fatty acid desaturase (FAD6) that is further desaturated to linolenic acid by the ω-3 fatty acid desaturase (FAD7/FAD8) (Shanklin and Cahoon, 1998; Hernández et al., 2011; Zhang et al., 2012).

After fatty acid synthesis in plastids, the fatty acyl-ACP moieties, mainly palmitoyl, stearoyl and oleoyl-ACPs are either used directly for lipid biosynthesis in the plastid or can be hydrolysed by fatty acyl-ACP thioesterases (FATA or FATB) to free fatty acids. They are later exported to ER in the form of acyl-CoA pool. The acyl-CoA pool undergoes several modifications regarding elongation, desaturation and exchange, catalyzed by a different ER membrane bound proteins, which constitute the eukaryotic pathway of lipid biosynthesis. In the ER, C18:1-CoA is incorporated into membrane phosphatidylcholine (PC) and desaturated to C18:2-PC by the ω-6 fatty acid desaturase (FAD2), and then C18:2-PC is desaturated to C18:3-PC by the ω-3 fatty acid desaturase (FAD3) (Bhunia et al., 2016). The first acylation reaction catalyzed by a glycerol-3-phosphate acyltransferase (GPAT) yields lysophosphatidic acid (LPA), which is further acylated by a lysophosphatidic acid acyltransferase (LPAAT) to the phosphatidic acid (PA). PA is then converted to 1, 2-sn-diacylglycerol (DAG) by the action of phosphatidic acid phosphatase (PAP). Finally triacylglycerol (TAG) is synthesized from DAG by 1, 2-sn-diacylglycerol acyltransferase (DGAT) followed by packaging into oil bodies with the aid of many integral proteins as reported in sesame (oleosin, caleosin, and steroleosin) (Baud and Lepiniec, 2010; Tzen, 2012).

### Plant Development

*FAD2* overexpression modifies many physiological features in transgenic seedlings, like seed germination and hypocotyl elongation, but such effects could not be observed after transformation with genes other than fatty acid desaturases (Wang et al., 2010). It was reported that *fad2* mutants formed dwarf phenotypes at a temperature of 22°C in Arabidopsis, when compared with the wild type plant (Miquel et al., 1993). The change in PUFA content due to *FAD2* mutation affects development in plants through the salicylic acid (SA), oxidase, abscisic acid, and jasmonic acid (JA) pathways (Martínez-Rivas et al., 2000; Kachroo A. et al., 2005; Regente et al., 2008). The cytochrome c oxidase expression was normally found inhibited in *A. thaliana* cells of the wild-type and mutant *fad2*, when exposed to a low temperature, unlike than reported in *FAD3* over expression lines (Martínez-Rivas et al., 2000). Kachroo A. et al. (2005) and Kachroo P. et al. (2005) reported that stearoyl-acyl carrier desaturase of *A. thaliana* mutants induced JA-responsive gene *PDF1.2* resistant to *Botrytis cinerea* pathogen, whereas Regente et al. (2008) observed major changes in the extracellular phospholipids, when sunflower seeds were treated with JA and ABA. Further, it was observed that the *fad2* mutant of *B. napus* has variable phenotypes, regarding leaf epidermal structure and permeability (Wang et al., 2003). This shows that the *FAD2* mutation is in charge for the changes in agricultural phenotypes of plants by affecting their development.

### Cold Tolerance

The skill of adjusting membrane fluidity by varying the unsaturated fatty acid contents is characteristic of cold-responsive plants (Upchurch, 2008). Under cold-stress conditions, FAD2 is of particular interest because of their modifying ability to increase unsaturated fatty acids (Falcone et al., 2004; Tang et al., 2005). The induction of Δ9 desaturase in *A. thaliana* (Kreps et al., 2002) and enhanced rate of *FAD2* gene expression in cotton was observed under cold stress environment (Kargiotidou et al., 2008). The cold stress in Arabidopsis also effected the transcription of the *FAD2* isogenes (*FAD2.2* and *FAD2.1*) (Gibson et al., 1994; Matsuda et al., 2005). Similar effect was observed in the olive drupes as there was an enhancement in *FAD2.2* and *FAD7* expression rate, and reduction in *FAD2.1* and *FAD6* in the first weeks of oil biogenesis (Matteucci et al., 2011). The mRNA levels of *FAD2-3* and *FAD2-4* increased, when temperatures were kept below the germination temperature (Kargiotidou et al., 2008). The treatment of cold stress also increased *FAD2.2* and *FAD7* mRNA levels in the epi-mesocarp cells of Canino and Moraiolo (Matteucci et al., 2011). The induction of other desaturase genes with the exception of *FAD8* and *FAD7*, which were induced under low-temperature conditions, was not reported in higher plants (Berberich et al., 1998; Falcone et al., 2004; Matsuda et al., 2005).

The Arabidopsis *fad6* mutant under chilling stress accumulated palmitoleic and oleic acids, and also thylakoid number was decreased in the chloroplast. In addition, the Arabidopsis *fad2* mutant has a low level of polyunsaturates in the extra chloroplast membrane lipids, and plant withering may result after long exposure to low temperatures (Miquel et al., 1993). In the *Synechocystis* sp., the mRNA levels of Δ6, Δ12, and ω-3 desaturases increased about 10-fold on reducing the temperature from 34°C to 22°C, in contrast, mRNA levels of Δ9-desaturase remained unchanged (Los et al., 1997). The difference in the *FAD2* expression patterns observed under cold stress conditions was correlated with the rise in unsaturated fatty acids, suggesting the direct role of the *FAD2* genes in membrane adaptation to cold stress.

### Salt Tolerance

One of the environmental stresses, such as salt induces the changes in fatty acid composition and many fatty acid desaturases participate in this event. It was reported that *FAD6* expression was responsive to osmotic and salt stress (Zhang J.T. et al., 2009). Tobacco plants transgenic with the antisense *FAD7* gene had low levels of polyunsaturated fatty acids, and were found to be more sensitive to drought and salt stress (Im et al., 2002). In contrast, heterologous expression of sunflower *FAD2-1* or *FAD2-3* in yeast had higher levels of dienoic fatty acids and thus showed increased yeast cell tolerance to salt (Rodríguez-Vargas et al., 2007). The polyunsaturated fatty acid composition in *fad2* mutants reduced the mobility of membrane lipids and as a result impaired the Na^+^/H^+^ pump function localized on the plasma membrane and tonoplast, when compared with vesicles isolated from control plants. In addition, the proton translocating activity of enzymes, such as V-ATPase, V-PPase and PM-ATPase was also reported lower in *fad2* mutants. It was now obvious that the role of Na^+^/H^+^ antiporters was inhibited in *fad2* mutants under salt stress conditions (Zhang et al., 2012). The *FAD2* gene thus played a key role in regulating and maintaining the lipid composition of intracellular membranes, biophysical characteristics, and proper functioning of membrane-attached proteins in salt stress conditions (Deuticke and Haest, 1987; Cooke and Burden, 1990; Zhang et al., 2012).

## Conclusion and Future Prospectus

Plants have many fatty acid desaturases, most of which are located in ER and chloroplast. FAD2 is an important desaturase enzyme, accountable for most of the polyunsaturated fatty acid synthesis in oilseed crops. The regulation of *FAD2* gene is important in understanding the composition of fatty acids and biosynthesis, plant development, and essential role in biotic and abiotic stresses like cold and salt tolerance. The changes in oil composition of edible seeds, especially the oleic and linoleic acid content can be modified genetically by silencing the *FAD* genes for a number of applications in industry, human health, and nutrition. High level of oleic acid in oil is one of the favored traits in oil engineering due to its high stability and several applications. Efforts taken for modifying plants genetically to produce pleasing unsaturated fatty acids had modest success. The identification of molecular markers for QTL can be designed for breeding high oleic varieties that could make possible the development of high or low oil content with the high oleic acid character. It is imperative to understand the mechanisms of regulation of fatty acid genes that will provide a base to modify the fatty acid compositions of membranes for the improvement of vitality and vigor of oilseed crops. Further study should also be focused on discovering other important microsomal oleate desaturase genes, and the regulation of enzymes at transcription and post-transcription levels. Effective strategies should be designed in plants to increase the accumulation of conjugated fatty acids. The approaches of metabolic engineering of oil traits can be an effective strategy for generating substantial levels of oil in crop plants.

## Author Contributions

AD has downloaded literature and written the manuscript and finally edited it. AC has also helped in writing the manuscript. PK has assisted in downloading the material for the manuscript. NA has given the idea of contents.

## Conflict of Interest Statement

The authors declare that the research was conducted in the absence of any commercial or financial relationships that could be construed as a potential conflict of interest.
